# C‐section increases cecal abundance of the archetypal bile acid and glucocorticoid modifying *Lachnoclostridium [clostridium] scindens* in mice

**DOI:** 10.14814/phy2.15363

**Published:** 2022-07-01

**Authors:** Sean H. Adams, Rachel Wright, Brian D. Piccolo, Becky Moody, James Sikes, Nathan Avaritt, Sree V. Chintapalli, Xiawei Ou

**Affiliations:** ^1^ Department of Surgery, School of Medicine University of California Davis California USA; ^2^ Center for Alimentary and Metabolic Science University of California Davis California USA; ^3^ Arkansas Children's Nutrition Center Little Rock Arkansas USA; ^4^ Department of Pediatrics University of Arkansas for Medical Sciences Little Rock Arkansas USA; ^5^ Department of Biochemistry University of Arkansas for Medical Sciences Little Rock Arkansas USA; ^6^ Department of Radiology University of Arkansas for Medical Sciences Little Rock Arkansas USA

**Keywords:** birth type, developmental programming, maternal microbiome, vaginal birth, xenobiotic

## Abstract

In humans and animal models, Cesarean section (C‐section) has been associated with alterations in the taxonomic structure of the gut microbiome. These changes in microbiota populations are hypothesized to impact immune, metabolic, and behavioral/neurologic systems and others. It is not clear if birth mode inherently changes the microbiome, or if C‐section effects are context‐specific and involve interactions with environmental and other factors. To address this and control for potential confounders, cecal microbiota from ~3 week old mice born by C‐section (*n* = 16) versus natural birth (*n* = 23) were compared under matched conditions for housing, cross‐fostering, diet, sex, and genetic strain. A total of 601 unique species were detected across all samples. Alpha diversity richness (i.e., how many species within sample; Chao1) and evenness/dominance (i.e., Shannon, Simpson, Inverse Simpson) metrics revealed no significant differences by birth mode. Beta diversity (i.e., differences between samples), as estimated with Bray‐Curtis dissimilarities and Aitchison distances (using log[*x* + 1]‐transformed counts), was also not significantly different (Permutational Multivariate ANOVA [PERMANOVA]). Only the abundance of *Lachnoclostridium* [*Clostridium] scindens* was found to differ using a combination of statistical methods (ALDEx2, DESeq2), being significantly higher in C‐section mice. This microbe has been implicated in secondary bile acid production and regulation of glucocorticoid metabolism to androgens. From our results and the extant literature we conclude that C‐section does not inherently lead to large‐scale shifts in gut microbiota populations, but birth mode could modulate select bacteria in a context‐specific manner: For example, involving factors associated with pre‐, peri‐, and postpartum environments, diet or host genetics.

## Graphical abstract

Abundances of cecal bacterial species found to be significantly altered by birth mode in 3 wk old male and female mice born naturally or via C‐section. (A) Lachnoclostridium [Clostridium] scindens was significantly higher in C‐Section mice. (B) Leptotrichia trevisanii abundances differed by birth mode, due to a sub‐set of natural birth animals positive for the microbe. Sample sizes are n=23 (natural birth, orange symbols) and n=16 (C‐section, blue symbols).

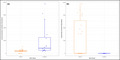

## 1 INTRODUCTION

In the last ~20 years the rate of Cesarean section (C‐section) delivery has rapidly increased across the globe, and in the U.S. has increased by 60% since 1996 (American College of Obstetricians and Gynecologists, Society for Maternal‐Fetal Medicine et al., 2014; Miseljic & Ibrahimovic, 2020). Recent data show that 31.7% of all births in the US (in 2019), over 40% in some Latin American countries, ~37% across Asia (Lumbiganon et al., 2010), and 18.6% on average globally were by C‐section (Betran, Ye, et al., 2016; Martin et al., 2021). When medically necessary, a C‐section can effectively prevent many instances of maternal or infant mortality. Experts convened by the World Health Organization to review the available literature have highlighted the medical importance of C‐section, but with the observation that “at [the] population level, C‐section rates higher than 10% are not associated with reductions in maternal and newborn mortality rates” ((Betran, Torloni, et al., 2016); also see (Betran et al., 2015)). While a C‐section is relatively safe, it is still major surgery and is associated with higher intra‐ and postpartum maternal odds for morbidities (e.g., at least one of: ICU admission, blood transfusion, hysterectomy, ileac artery ligation) and perinatal risks (e.g., NICU admissions), which could in theory have implications on future pregnancies as well as long‐term effects for the mother and offspring (Lumbiganon et al., 2010; Souza et al., 2010). The World Health Organization recently reassessed available evidence and concluded that C‐section delivery should ideally be undertaken only when medically necessary, acknowledging that the relationship between C‐section and long‐term pediatric outcomes is unclear (World Health Organization, 2015).

Developmental impacts of C‐section on the offspring remain to be elaborated, but could involve multiple physiological systems including immune, metabolic, and behavioral/neurologic. Regarding the latter, while some studies have shown no significant difference in intelligence quotient (Khadem & Khadivzadeh, 2010), others have found associations between C‐section and an increased risk for autism spectrum disorder and/or attention deficit/hyperactivity disorder (Amiri et al., 2012; Curran, Dalman, et al., 2015; Curran, O'Neill, et al., 2015; Talge et al., 2016). We have demonstrated that 2‐week old infants born by C‐section had lower brain white matter integrity and less functional connectivity across different brain regions compared to infants born by natural delivery (Deoni et al., 2019). The effects on white matter remained through at least 3 year of age; thus, while this effect may be transient, these results highlight that birth mode may influence neurodevelopmental outcomes in offspring. Other data point to potential links between birth mode and body composition: One study reported two‐fold higher risk for adolescent obesity in children born by C‐section in the United Kingdom (Blustein et al., 2013). As recently reviewed by Faúndes et al. (2021), most but not all studies have demonstrated modestly higher odds for childhood or young adult obesity in C‐section offspring. A large systemic review and meta‐analysis found that children delivered by C‐section had higher risk for developing respiratory tract infections, obesity, and manifestations of asthma significantly compared to children delivered vaginally, but there were inconclusive results regarding the risk of developing diabetes mellitus type 1 or neurological disorders (Slabuszewska‐Jozwiak et al., 2020). The underlying mechanisms that could lead to differential physiological outcomes in C‐section compared to natural birth offspring are still unknown. We have hypothesized that innate differences in gut microbiota are involved in brain differences (Deoni et al., 2019), and it has also been speculated that the gut microbiome contributes to C‐section‐associated risks for obesity (Faundes et al., 2021) and asthma (Stokholm et al., 2020).

The hypothesis that birth mode‐specific gut microbiota populations exist is logical based on the fact that C‐section deliveries interrupt the exposure of the newborn to the maternal vaginal microbiota. Natural birth involves neonatal exposure to the vaginal microbiota, which, in theory, leads to distinct maternally‐derived microbiota patterns in babies, some of which could be long‐lasting. In contrast, C‐section offspring patterns would derive initially from “environmental sources” only. Indeed, using fecal cultures to characterize temporal patterns of bacteria in infants aged up to 180 days, colonization appeared delayed in children born by C‐section with pre‐delivery antibiotic prophylaxis for their mothers (Gronlund et al., 1999). Penders et al. (2006) reported that in 1 mos old C‐section infants, there was lower fecal prevalence of *Bifidobacteria* and *Bacteroides* as measured by polymerase chain reaction, findings similar to Huurre et al. (2008) who used fluorescence in situ hybridization methods. In babies aged 2 and 4 days, a significantly different fecal microbiome profile was reported comparing vaginal to C‐section birth: The latter had lower *Bacteroides*, *Bifidobacteria*, and *E. coli* and higher relative abundances of *Staphylococcus* spp., *Clostridium* spp. and *Enterbacter* spp. (Liu et al., 2015). Some studies have found that differences in the gut become insignificant after 6 weeks (Chu et al., 2017), but other results support the notion that differences in birth mode affects select gut microbiota for a longer time frame. For instance, *Bacteriodetes* phyla were lower at least through 12 mos of age in children born to C‐section in one study (Jakobsson et al., 2014). At 1 year postpartum, children born by C‐section retained the reduced *Bacteroides* also seen at 1 week and 1 mos, but the lower *Bifidobacteria* of infancy had normalized (Stokholm et al., 2020). Using fecal samples from 7 year olds with vaginal or C‐section births, Salminen et al. (2004) used a fluorescence in situ hybridization method with probes specific to *Bifidobacteria* spp., *Lactobacilli/Enterococci* spp., *Bacteriodes*, and *Clostridia*; the latter class was significantly lower in children born by C‐section. Phylum‐level differences in feces were observed as early as 7 days postpartum for C‐section infants (e.g., lower relative abundances of Actinobacteria and Bacteroidetes and higher relative Firmicutes), which was still observed in samples at 31 days (Selma‐Royo et al., 2020).

Despite accumulating evidence that birth mode impacts the gut microbiota and functional outcomes in humans, interpretations may be uncertain due to the involvement of numerous confounding factors (i.e., home environment, host genetics, infant nutrition, weaning foods and timing, etc.). Thus, the field awaits a definitive answer to the question, “Does birth mode, by definition, inherently lead to a difference in the gut microbiome?” Considering the potential significance of this to public health and clinical practice, it is imperative to answer this question through studies designed to examine the effects of birth mode while minimizing confounders. To this end, we conducted a controlled mouse model experiment to observe whether C‐section affects the cecal microbiota when compared to offspring derived from vaginal birth. We reasoned that if birth mode inherently leads to significant differences in cecal microbiota, this would be detectable in young animals tested at the ~3 weeks postpartum, peri‐weaning stage.

## 2 METHODS

### 2.1 Animals and C‐section model

Studies were approved by the UAMS Animal Use Committee (Protocol #3740). Timed pregnant CD‐1 IGS mice (strain code 022) were supplied by Charles River. First‐time pregnant mothers 8–10 weeks of age were timed bred to arrive on embryonic day 4 and day 7 (EMD4, EMD7). The dams were single‐housed in cages with TEK‐Fresh laboratory animal bedding (Envigo‐Teklad 7099) and a single nestlet (NES 3600, Ancare), and fed ad libitum rodent chow (Envigo‐Teklad 8640) and water. The vivarium light cycle was 12 h:12 h, and the housing temperature 22°C. EMD7 dams served as foster dams and had offspring at gestation day 19. EMD4 dams served as the source for naturally‐born and C‐section offspring. On gestation day 19, a subset of EMD4 dams (*n* = 3) were anesthetized for C‐section with isoflurane (oxygen flow 1.0 LPM and mix of 2.5%–3.5%). For this protocol, the dam was laid on its back, the abdomen shaved and cleaned with alcohol and betadine. An incision was made the full length of the abdomen to gain access to the peritoneal cavity. The uterine horn was pulled out and laid on a warmed saline‐moistened gauze pack. The horn was cut along the whole length with scissors, along the side opposite of the placenta. Pups were pulled away from their connections with the uterus so the complete fetus with amniotic sac and placenta were removed. The pups were removed from their amniotic sacs and gently wiped clean and dried with warmed gauze. Pups were then placed on a heated pad (Lectro‐Kennel Model 1000, K & H Manufacturing) until dry, warm, pink and beginning to move. Pups were then transferred to a CD‐1 foster dam after her entire litter had just been removed. C‐section offspring were weighed two consecutive postnatal days (PND1, PND2) to confirm positive weight gain. The offspring from natural delivery dams (*n* = 4) offspring were cross‐fostered approximately 1 day after parturition (placed with recipient dam at ~10:00 the day after parturition) and weighed on PND2. C‐section dams cross‐fostered 14 males/18 females to three EMD7 dams, with a total of four deaths during PND1. Natural‐birth dams cross‐fostered 25 males/19 females to four EMD7 dams, with no deaths. Typical cross‐fostered litters were 11 or 12 pups, except one cross‐fostered C‐section litter had 10 (due to a single pup PND1 death) and one cross‐fostered natural born litter had 17.

### 2.2 Tissue and biospecimen collections

Cross‐fostered offspring tissues were collected on PND21/22. Each mouse was weighed and then euthanized under CO_2_. Whole cecum tissues were collected and placed in liquid nitrogen until storage at −80°C. These procedures were initiated at ~09:00 and ended at ~14:30 on each collection day, with treatment groups mixed throughout the day in terms of collection time.

### 2.3 Microbiome sequence analysis

Previously collected cecum tissues remained on dry ice until individual samples were thawed just enough to remove from storage tubes. Each sample was then placed on a sterilized glass plate and a sterile disposable scalpel was used to create an incision. A sterile disposable spatula was used to transfer cecal contents into a sterile 1.5 ml polypropylene tube on dry ice. DNA was extracted from approximately 150–200 mg of cecal contents using the QIAamp Fast Stool minikit protocol (Qiagen) following the manufacturer's instructions. For each sample, total DNA concentration, purity, and quality were measured using the AATI Fragment Analyzer (Agilent). Extracted DNA was stored at −80°C until library preparation. DNA libraries were prepared using the Nextera DNA Library Prep kit (Illumina) followed by shotgun sequencing performed on an Illumina NextSeq 500 platfrom with a 2 × 150‐bp, paired‐end run. Raw sequence data files were demultiplexed and converted into FASTQ files using Casava v.1.8.2 (Illumina). MEGAN Ultimate Edition software (v6.18) was used to analyze the reads by performing taxonomic binning and assigning reads to respective nodes in the National Center for Biotechnology Information (NCBI) taxonomy (Huson et al., 2016). Ultrafast alignment of reads against NCBI‐nr (non‐redundant) was achieved by the program DIAMOND v0.9.9.110 (Buchfink et al., 2015). Furthermore, Meganizer (an integral component of MEGAN software) was used in performing taxonomic analysis.

### 2.4 Microbial taxonomy analysis and statistics

Alpha‐diversity measures were calculated using the estimated richness (McMurdie & Holmes, 2013) function from the phyloseq package (v 1.36.0) and included the metrics: Observed total species, Chao1, Simpson, and Inverse Simpson. The differences of each metric between the two birth modes were assessed with a Mann–Whitney *U* test. Beta‐diversity was assessed with Bray‐Curtis Dissimilarities and Aitchison distances (i.e., Euclidian distances between centered log‐ratio transformed counts) using log (*x* + 1)‐transformed counts, visualized with Principal Co‐ordinate Analysis (PCoA). Birth mode differences in beta‐diversity was determined with Permutational Multivariate Analysis of Variance (PERMANOVA). Analyses of bacterial species differences were performed using the ALDEx2 v1.24 (Fernandes et al., 2014), DESeq2 v1.32 (Love et al., 2014; McMurdie & Holmes, 2014), and metagenomeSeq v1.34 (Paulson et al., 2013) pipelines, while identifying overlapping significant taxa. We used multiple methods due to the known incongruences between statistical approaches for differential abundance analysis of microbial sequencing data (Weiss et al., 2017). *p*‐values obtained from sequencing data analyses were corrected for multiple comparisons using the Benjamini‐Hochberg method and considered significant at false discovery rate (FDR) < 0.05. All statistical analyses of microbial sequencing data were conducted in the R Statistical Language (v 4.1.0). Sequencing files are publicly available on the National Center for Biotechnology Information Sequence Read Archive repository with the accession number PRJNA841368.

## 3 RESULTS

### 3.1 Body mass

At PND2, average body weight was slightly (~12%) but significantly higher in natural‐born compared to C‐section cross‐fostered pups (1.8 ± 0.03 and 1.6 ± 0.03 g, respectively; *p* < 0.0001). In a subset of mice (used in separate studies) measured at PND 7/8, this relative difference remained in natural‐born versus C‐section pups (5.6 ± 0.12 g [*n* = 21] vs. 4.9 ± 0.14 g [n = 16], respectively; *p* < 0.01). However, by PND 21/22, in the sub‐group of mice used for metagenome analyses herein, body weights no longer differed (15.5 ± 0.35 g [*n* = 21] vs. 15.7 ± 0.24 g [*n* = 16], respectively; *p* = 0.68).

### 3.2 Cecal content microbiota populations in natural born versus C‐section pups

There were 39 samples with 16 total mice born via C‐section and 23 from natural vaginal birth. Of those mice, 19 were female and 20 were male. A total of 601 unique species were observed across all samples. All results for statistical analyses are provided in Table S1 deposited as DOI: https://doi.org/10.15482/USDA.ADC/1524407 (USDA Ag Data Commons: https://doi.org/10.15482/USDA.ADC/1524407).

**FIGURE 1:**
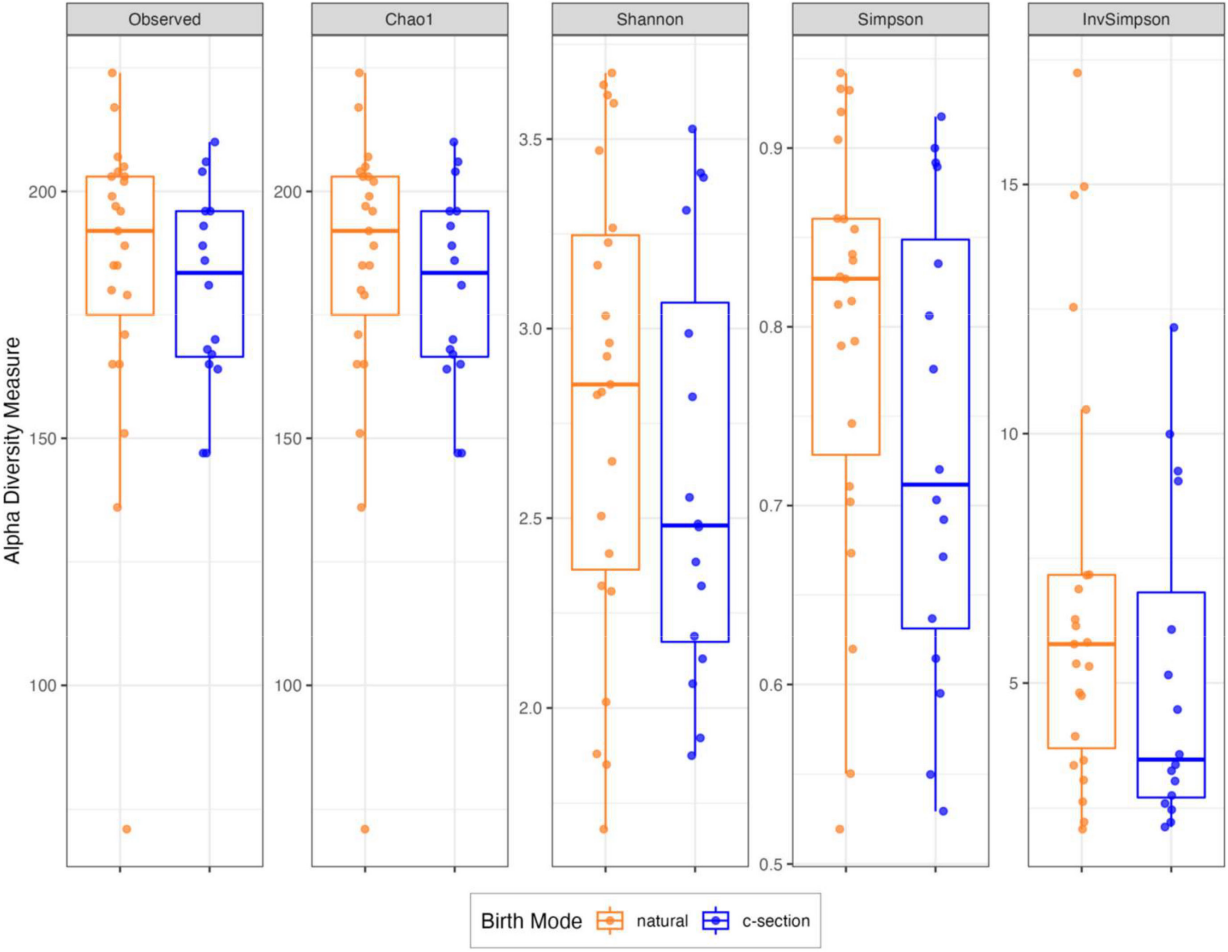
Indices of α‐diversity do not significantly differ in cecal microbiota populations derived from 3 week old male and female mice born naturally or via C‐section. Sample sizes are *n* = 23 (natural birth, orange symbols) and *n* = 16 (C‐section, blue symbols).

**FIGURE 2:**
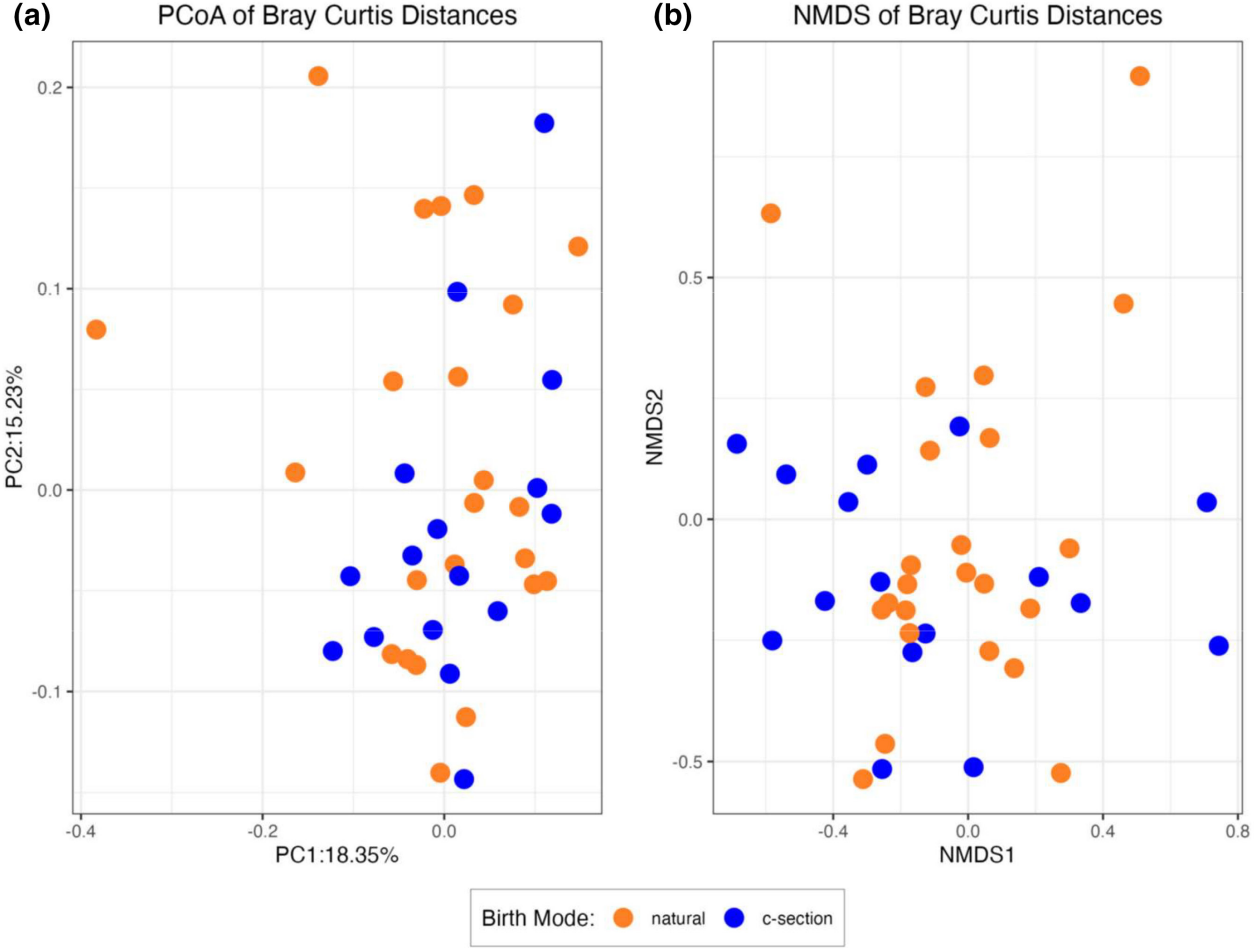
Indices of β‐diversity do not differ in cecal microbiota populations derived from 3 week old male and female mice born naturally or via C‐section. Sample sizes are *n* = 23 (natural birth, orange symbols) and *n* = 16 (C‐section, blue symbols).

Alpha diversity metrics of richness and evenness are presented in Figure 1. No statistical differences were found in any alpha diversity measurement between mice born via C‐section and those born from natural vaginal birth. Visualization of beta diversity (Bray‐Curtis Dissimiliarities) using Principal Co‐ordinate Analysis (PCoA) and Non‐metric Dimensional Scaling (NMDS) are presented on Figure 2. Significant overlap of samples was visually apparent on Components 1 and 2 axes, suggesting little variation associated with birth mode. This was corroborated with a lack of statistical significance in PERMANOVA analyses for both Bray‐Curtis Dissimilarities (*p* = 0.094) and Aitchinson distances (*p* = 0.089).

**FIGURE 3:**
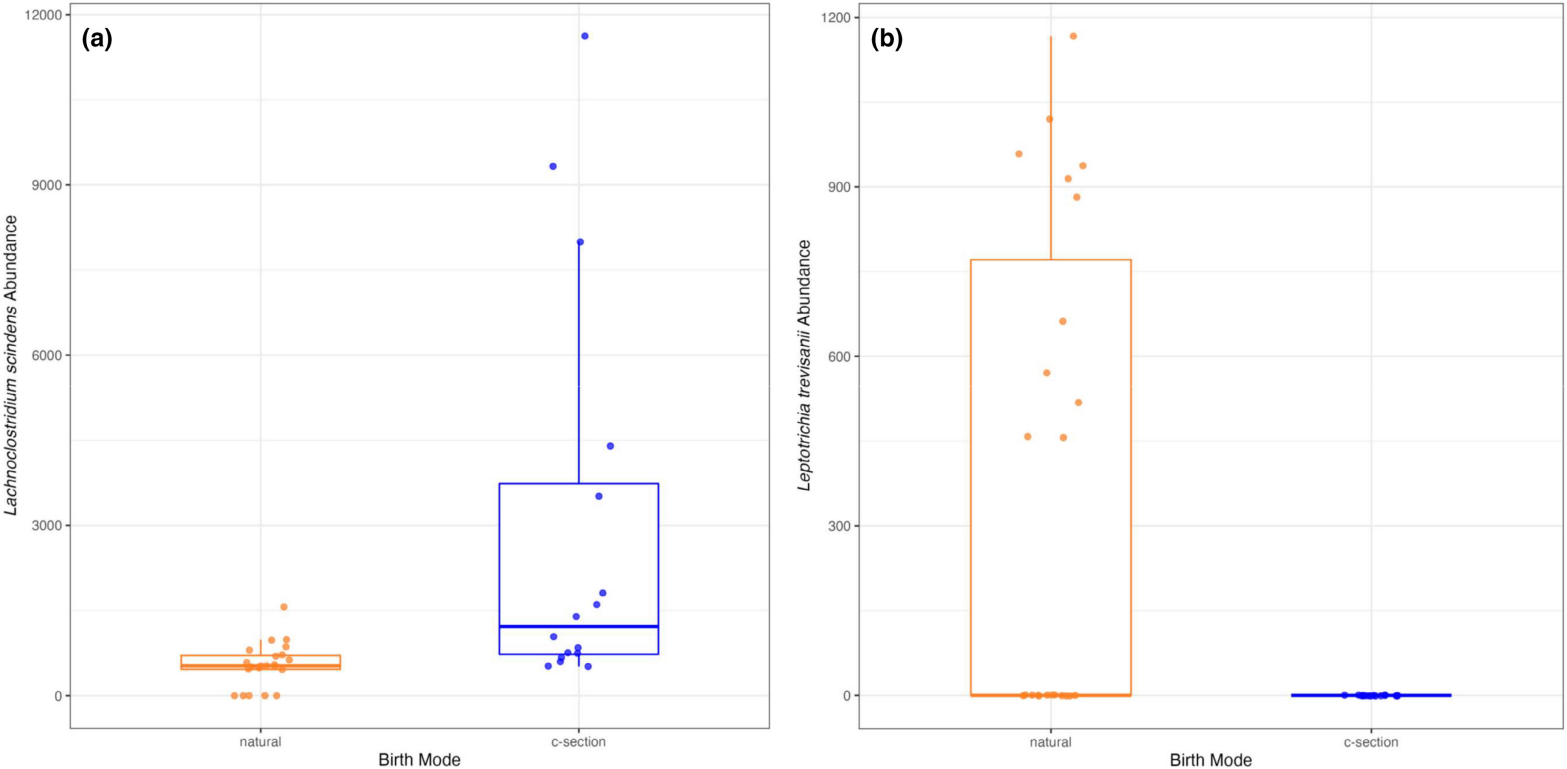
Abundances of cecal bacterial species found to be significantly altered by birth mode in 3 week old male and female mice born naturally or via C‐section. (a) *Lachnoclostridium [clostridium] scindens*: In both ALDEx2 (*p* = 0.01) and DESeq2 (*p* = 0.001) statistical pipelines, the microbe prevalence was significantly higher in C‐section mice versus natural birth mice. (b) *Leptotrichia trevisanii*: Abundances differed by birth mode, due to a sub‐set of natural birth animals positive for the microbe; DESeq2 (*p* < 0.001) and metagenomeSeq (*p* = 0.004) statistical pipelines were significant. The microbe was not present in cecal contents of C‐Section mice in contrast to natural‐born mice where presence was detected about half of the animals. Sample sizes are *n* = 23 (natural birth, orange symbols) and *n* = 16 (C‐section, blue symbols).

To determine differentially expressed species, we used a conservative approach that required that any given taxon be significant in at least 2 of the 3 applied statistical tests. In both ALDEx2 and DESeq2 statistical pipelines, the Class Clostridia microbe *Lachnoclostridium [Clostridium] scindens* was significantly higher in C‐section mice versus natural birth mice (Figure 3a). In the DESeq2 and metagenomeSeq statistical pipelines, the Class Fusobacterii bacterium *Leptotrichia trevisanii* was found to be significantly different by birth mode: Not present in cecal contents of C‐Section mice in contrast to natural‐born mice where presence was detected about half of the animals (Figure 3b). The latter phenomenon was seen across foster litters and was not different by sex. Together with the diversity measures, the results overall indicate that there was not a major difference in cecal microbiota populations attributable to birth mode in this mouse model.

## 4 DISCUSSION

Several studies in humans and animal models suggest that birth mode impacts the composition of the gut microbiome, but not all results are consistent in this regard. Furthermore, an unresolved issue is whether C‐section inherently leads to a shift in the offspring's gut microbe population, or if birth mode effects come about through a more complex association with environmental or other factors that change during development. To address these questions, we took advantage of an experimental C‐section mouse model where aspects such as housing, postnatal diet, and strain are fully controlled (and matched to natural birth animals). Environmental confounders were further minimized by examining the microbiome at an early age, just prior to weaning. The results indicated that few differences in microbiota populations were apparent at PND 21/22, highlighting that birth mode alone may not fully explain reported effects of C‐section on offspring gut microbiome. It is acknowledged that this perspective stems from a single age analysis, and we cannot discount the possibility that birth mode impacts microbiome more profoundly at different life stages.

The largely “null” result in the current study with respect to C‐section shifts in gut microbiota contrasts with several papers reporting birth mode microbiota differences in mouse models. For example, behavioral changes in C‐section mice have been hypothesized to be linked to differences in specific gut microbiota. In one study, lower relative abundance of fecal *Bifidobacterium* spp. in male C‐section NIH Swiss mice (at 3 weeks) was detected, and there were also early‐life social, cognitive and anxiety differences (Morais et al., 2020). Some behavioral deficits of C‐section (e.g., social novelty recognition, anxiety/marble burying) persisted into later life, but could be dampened in part or completely mitigated by co‐housing with vaginal birth animals, or through dietary maneuvers to increase Bifidobacteria. In 8 week old C‐section C57BL/6 mice, there were reductions in exploratory behavior, higher anxiety‐like behaviors, and deficits in sociability indices especially in females, (Zachariassen et al., 2021). The ANCOM test revealed a higher relative abundance of *Prevotella* spp. (C‐section, 8.4%; vaginal delivery, 0.9%) and a lower abundance of *Candidatus arthromitus* (C‐section, 0.09%; vaginal delivery, 0.55%) in C‐section mice. The investigators speculated that differences in the gut microbiome measured at age 5 weeks may have contributed to behavioral phenotypes. Aspects that could make study‐to‐study comparisons challenging are the differing types of biospecimens (i.e., cecal vs. ileal vs. fecal), analysis methods (i.e., 16S vs. metagenomic), feeding regimens, and ages that can be employed across experiments.

Other experiments have demonstrated differences in immune function due to C‐section, which has been attributed in part to differences in the gut microbiota activities. Hansen et al. (2014) reported that 10–30 week old male and female mice delivered by C‐section had lower relative amounts of Foxp3‐positive regulatory T‐cells, CD103‐positive dendritic cells and reduced mRNA expression of the anti‐inflammatory *IL‐10* in lymph nodes and spleen. While no birth mode‐associated differences were seen in adult microbiota, the C‐section mice around weaning had significantly lower relative abundances of *Rikenellaceae* and *Ruminococcus* plus higher *Bacteroides acidifaciens* and *Lachnospiraceae* in feces when compared to vaginal birth mice (Hansen et al., 2014). Zachariassen et al. reported that 8 week old C57BL/6 C‐section mice had lower regulatory T cells, higher invariant NKT (iNKT) cells, and displayed altered ileal mRNA expression of immune and inflammation markers compared to vaginal birth mice: For example, *lower Foxp3, IL‐10, Ctla4, Cd11c, Egr2, Nos2*, and higher iNKT markers *IL‐4, IL‐15*) (Zachariassen, Krych, et al., 2019). Several aspects were phenocopied in germ‐free mice inoculated with feces preparations from C‐Section mice. In an oxazolone‐induced colitis model in 8 week old C57BL/6 mice, C‐section mice had more severe reactions to the challenge: For example, greater body weight and colon tissue weight losses, higher colon TNF‐α and colon immune cell infiltration, lower expression of the gut barrier‐associated genes occludin and tight junction protein 1 (Zachariassen, Hansen, et al., 2019). In that study, several colitis‐like symptoms (e.g., greater gut permeability, colon immune cell infiltration) were observed in germ‐free mice following inoculation with a feces preparation from 9 week old colitis‐free C‐section donor mice, but this was not observed using inoculate derived from naturally‐born mice.

The specific mechanisms and molecular messages that impart C‐section‐associated phenotypes to offspring remain to be established and could in theory come about through changes in functional characteristics of gut microbes, even in the absence of major population shifts. Interestingly, our analysis revealed a differential abundance in *Lachnoclostridium [Clostridium] scindens* (a.k.a. *C. scindens*) that was significantly increased in cecum from C‐section offspring. This species is an archetypal bile acid modifying microbe that expresses the enzyme 7α‐hydroxylase that drives conversion of cholic acid to deoxycholic acid (DCA; a.k.a. 7‐oxodeoxycholic acid) and 7β‐hydroxylase that catalyzes conversion of DCA to 7‐epicholic acid (Ridlon et al., 2006). This microbe also expresses a cortisol‐regulated desABCD operon containing genes involved in glucocorticoid metabolism: That is, 20α‐hydroxysteroid dehydrogenase involved in conversion of cortisol to androgens and steroid‐17,20‐desmolase that forms pro‐androgens (Devendran et al., 2018; Ridlon et al., 2013). *C. scindens* has also been shown to secrete antibacterial factors (e.g., [Kang et al., 2019]). These and other potential bioactivities support the speculation that birth mode‐associated differences in *Lachnoclostridium [Clostridium] scindens* can modify hormone and bile acid status, in turn altering host physiology in response to C‐section delivery. We also detected a difference in abundance patterns of *Leptotrichia trevisanii* (none detected in C‐section mice). Bacteremia associated with this “opportunistic” microbe has been reported and seems to manifest primarily in the immunocompromised condition (e.g., see: [Inal & Hazirolan, 2021; Schrimsher et al., 2013; Tee et al., 2001]). However, considering only half of the natural birth mice harbored this microbe, the functional ramification of the differential cecal abundance of *L. trevisanii* due to birth mode is not clear.

Strengths of the current experiment include a large sample size per treatment group, a well‐matched set of housing and other conditions in C‐section versus natural birth mice, and a comprehensive metagenomics‐based evaluation of the cecal microbiome. The findings were robust with respect to the assessment of microbiota in CD‐1 IGS mice in the peri‐weaning period, but which aspects translate to human infants remain to be validated. Our experiments only studied a single age in early development in one mouse strain, and thus were not designed to evaluate the potential impacts of genetics or various housing conditions, postweaning diet, and other environmental factors that might interact with birth mode to modify microbiota. It is also acknowledged that since the analyses focused on the lower gut cecal contents, bioregional shifts in the microbiome, if any, were not addressed. Finally, the approach focused on the measurement of taxonomic changes due to birth mode and not potential microbe functional differences such as xenometabolism or production of signaling molecules. Despite these limitations, the results support the conclusion that C‐section does not inevitably lead to large‐scale shifts in the microbiota population, suggesting that associations of C‐section and the microbiome are context‐specific and may involve additional players such as the prepartum/peripartum/postpartum environment, diet or genetics.

## AUTHORS' CONTRIBUTIONS

Xiawei Ou, Brian D. Piccolo, James Sikes, Sean H. Adams: Conceived and developed the research plan; Xiawei Ou, James Sikes: Coordinated and conducted the birth mode model studies and biospecimen collection; Brian D. Piccolo, Becky Moody, Sree V. Chintapalli: Analyzed the microbiome data and performed the statistical analysis; Nathan Avaritt, performed DNA sequencing assays; Rachel Wright, Xiawei Ou, Sean H. Adams: Wrote the manuscript with input from all authors; all authors have responsibility for final content and read and approved the final manuscript.

## FUNDING INFORMATION

This research was supported by funding from the Arkansas Bioscience Institute (to XO). Additional support was from USDA‐ARS Project 6026–51000‐010‐05S.

## CONFLICT OF INTEREST

Sean H. Adams is the founder and principal of XenoMed, LLC, which is focused on research and discovery in the area of microbial metabolism. XenoMed had no part in the research design, funding, results or writing of the manuscript.

## Supporting information




Table S1
